# Crystal structure of 1-meth­oxy-2,2,2-tris­(pyrazol-1-yl)ethane

**DOI:** 10.1107/S1600536814018789

**Published:** 2014-08-23

**Authors:** Ganna Lyubartseva, Sean Parkin, Morgan D. Coleman, Uma Prasad Mallik

**Affiliations:** a Department of Biochemistry and Chemistry, Southern Arkansas University, Magnolia, AR 71753, USA; b Department of Chemistry, University of Kentucky, Lexington, KY 40506, USA

**Keywords:** crystal structure, tris­(pyrazol-1-yl)ethane, scorpionate ligands

## Abstract

The title compound, C_12_H_14_N_6_O, consists of three pyrazole rings bound *via* nitro­gen to the distal ethane carbon of meth­oxy ethane. The dihedral angles between the three pyrazole rings are 67.62 (14), 73.74 (14), and 78.92 (12)°. In the crystal, mol­ecules are linked by bifurcated C—H,H⋯N hydrogen bonds, forming double-stranded chains along [001]. The chains are linked *via* C—H⋯O hydrogen bonds, forming a three-dimensional framework structure. The crystal was refined as a perfect (0.5:0.5) inversion twin.

## Related literature   

For properties of pyrazole-based tridentate ligands, see: Paulo *et al.* (2004 ▶); Bigmore *et al.* (2005 ▶). For nickel and cobalt complexes of N-donor tridentate scorpionate ligands, see: Lyubartseva *et al.* (2011 ▶, 2012 ▶, 2013*a* ▶,*b* ▶); Lyubartseva & Parkin (2009 ▶). For the synthesis of the title compound, see: Maria *et al.* (2007 ▶).
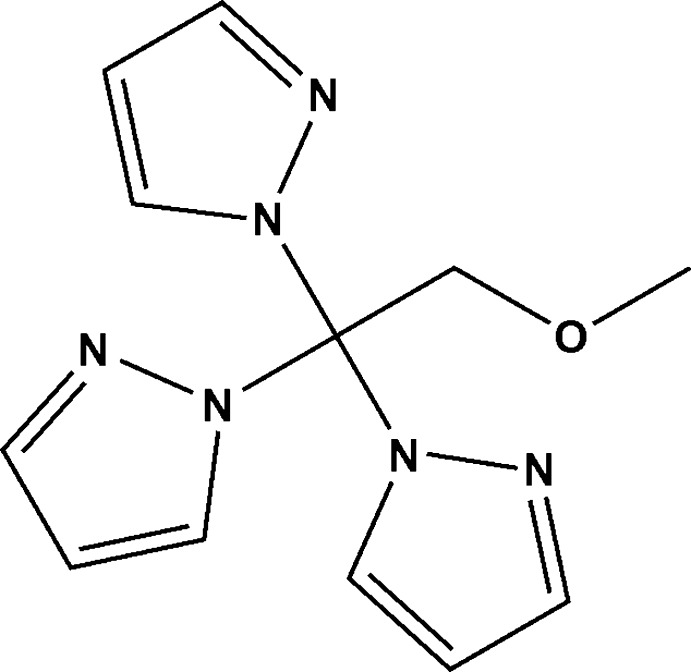


## Experimental   

### Crystal data  

C_12_H_14_N_6_O
*M* *_r_* = 258.29
Monoclinic, 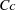

*a* = 12.5828 (3) Å
*b* = 12.3847 (3) Å
*c* = 8.4807 (2) Å
β = 102.5635 (11)°
*V* = 1289.94 (5) Å^3^
*Z* = 4
Mo *K*α radiation
μ = 0.09 mm^−1^
*T* = 90 K
0.28 × 0.20 × 0.16 mm

### Data collection  

Nonius KappaCCD diffractometer
Absorption correction: multi-scan (*SADABS*; Sheldrick, 1996 ▶) *T*_min_ = 0.749, *T*_max_ = 0.942
11397 measured reflections
2934 independent reflections
2386 reflections with *I* > 2σ(*I*)
*R*_int_ = 0.032

### Refinement  

*R*[*F* ^2^ > 2σ(*F* ^2^)] = 0.042
*wR*(*F* ^2^) = 0.102
*S* = 1.10
2934 reflections
174 parameters
2 restraints
H-atom parameters constrained
Δρ_max_ = 0.23 e Å^−3^
Δρ_min_ = −0.18 e Å^−3^
Absolute structure: Refined as a perfect (*i.e.* 50:50) inversion twin

### 

Data collection: *COLLECT* (Nonius, 1998 ▶); cell refinement: *SCALEPACK* (Otwinowski & Minor, 1997 ▶); data reduction: *DENZO-SMN* (Otwinowski & Minor, 1997 ▶); program(s) used to solve structure: *SHELXS97* (Sheldrick, 2008 ▶); program(s) used to refine structure: *SHELXL2014* (Sheldrick, 2008 ▶); molecular graphics: *XP in *SHELXTL** (Sheldrick, 2008 ▶); software used to prepare material for publication: *SHELXL2014* and *PLATON* (Spek, 2009 ▶).

## Supplementary Material

Crystal structure: contains datablock(s) global, I. DOI: 10.1107/S1600536814018789/su2774sup1.cif

Structure factors: contains datablock(s) I. DOI: 10.1107/S1600536814018789/su2774Isup2.hkl

Supporting information file. DOI: 10.1107/S1600536814018789/su2774Isup3.cml

**Fig. 1.**
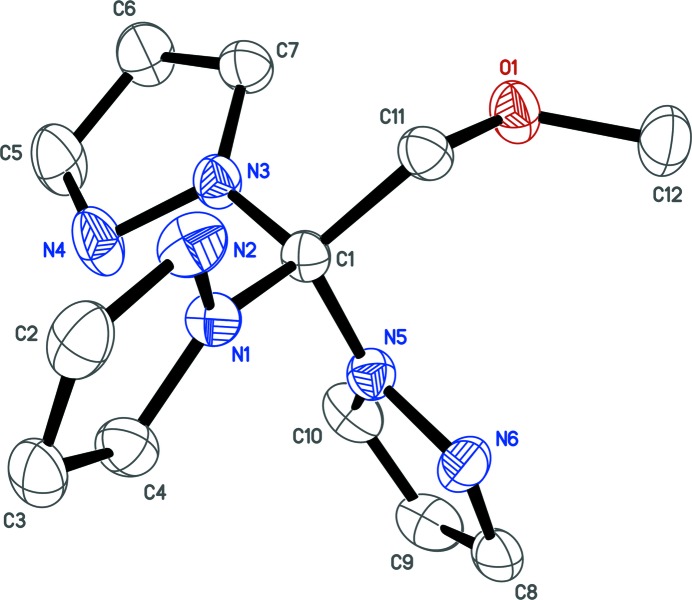
View of molecular structure of the title molecule, with atom labelling. Displacement ellipsoids are drawn at the 50% probability level. H atoms have been omitted for clarity.

. DOI: 10.1107/S1600536814018789/su2774fig1.tif
View of mol­ecular structure of the title mol­ecule, with atom labelling. Displacement ellipsoids are drawn at the 50% probability level. H atoms have been omitted for clarity.

CCDC reference: 1019968

Additional supporting information:  crystallographic information; 3D view; checkCIF report

## Figures and Tables

**Table 1:** Hydrogen-bond geometry (Å, °)

*D*—H⋯*A*	*D*—H	H⋯*A*	*D*⋯*A*	*D*—H⋯*A*
C5—H5*A*⋯N2^i^	0.95	2.51	3.453 (4)	171
C9—H9*A*⋯N2^ii^	0.95	2.61	3.433 (4)	145
C4—H4*A*⋯O1^iii^	0.95	2.53	3.444 (4)	162

Symmetry codes: (i) 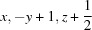; (ii) 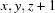; (iii) 
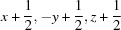
.

## supplementary crystallographic information

### S1. Synthesis and crystallization

The title compound was prepared using the published procedure (Maria *et al.*, 2007). Colourless block-like crystals were obtained by slow evaporation of a di­ethyl ether solution of pure product. Spectral and other characterizations are in good accordance with the previously reported data (Maria *et al.*, 2007).

### S2. Refinement

H atoms were located in difference Fourier maps, but were subsequently included in the refinement using a riding model approximation: C—H = 0.95 - 0.99 Å with U_iso_(H) = 1.5*U*_eq_(C-methyl) and = 1.2*U*_eq_(C) for other H atoms. The crystal was refined as a perfect (0.5:0.5) inversion twin.

### Crystal data

**Table:**

C_12_H_14_N_6_O	*F*(000) = 544
*M**_r_* = 258.29	*D*_x_ = 1.330 Mg m^−^^3^
Monoclinic, *C**c*	Mo *K*α radiation, λ = 0.71073 Å
*a* = 12.5828 (3) Å	Cell parameters from 1549 reflections
*b* = 12.3847 (3) Å	θ = 1.0–27.5°
*c* = 8.4807 (2) Å	µ = 0.09 mm^−^^1^
β = 102.5635 (11)°	*T* = 90 K
*V* = 1289.94 (5) Å^3^	Block, colourless
*Z* = 4	0.28 × 0.20 × 0.16 mm

### Data collection

**Table:**

Nonius KappaCCD diffractometer	2934 independent reflections
Radiation source: fine-focus sealed-tube	2386 reflections with *I* > 2σ(*I*)
Detector resolution: 9.1 pixels mm^-1^	*R*_int_ = 0.032
φ and ω scans at fixed χ = 55°	θ_max_ = 27.5°, θ_min_ = 2.3°
Absorption correction: multi-scan (*SADABS*; Sheldrick, 1996)	*h* = −16→16
*T*_min_ = 0.749, *T*_max_ = 0.942	*k* = −16→16
11397 measured reflections	*l* = −10→11

### Refinement

**Table:**

Refinement on *F*^2^	Hydrogen site location: difference Fourier map
Least-squares matrix: full	H-atom parameters constrained
*R*[*F*^2^ > 2σ(*F*^2^)] = 0.042	*w* = 1/[σ^2^(*F*_o_^2^) + (0.0453*P*)^2^ + 0.5917*P*] where *P* = (*F*_o_^2^ + 2*F*_c_^2^)/3
*wR*(*F*^2^) = 0.102	(Δ/σ)_max_ < 0.001
*S* = 1.10	Δρ_max_ = 0.23 e Å^−^^3^
2934 reflections	Δρ_min_ = −0.18 e Å^−^^3^
174 parameters	Extinction correction: *SHELXL2014* (Sheldrick, 2008), Fc^*^=kFc[1+0.001xFc^2^λ^3^/sin(2θ)]^-1/4^
2 restraints	Extinction coefficient: 0.0127 (19)
Primary atom site location: structure-invariant direct methods	Absolute structure: Refined as a perfect (*i.e.* 50:50) inversion twin.
Secondary atom site location: difference Fourier map	

### Special details

**Table:**

Experimental. The crystal was mounted with polyisobutene oil on the tip of a fine glass fibre, which was fastened in a copper mounting pin with electrical solder. It was placed directly into the cold gas stream of a liquid nitrogen based cryostat, according to published methods (Hope, 1994; Parkin & Hope, 1998).Diffraction data were collected with the crystal at 90 K, which is standard practice in this laboratory for the majority of flash-cooled crystals.
Geometry. All e.s.d.'s (except the e.s.d. in the dihedral angle between two l.s. planes) are estimated using the full covariance matrix. The cell e.s.d.'s are taken into account individually in the estimation of e.s.d.'s in distances, angles and torsion angles; correlations between e.s.d.'s in cell parameters are only used when they are defined by crystal symmetry. An approximate (isotropic) treatment of cell e.s.d.'s is used for estimating e.s.d.'s involving l.s. planes.
Refinement. Refinement progress was checked using *PLATON* (Spek, 2009) and by an *R*-tensor (Parkin, 2000). The final model was further checked with the IUCr utility *checkCIF*.

### Fractional atomic coordinates and isotropic or equivalent isotropic displacement parameters (Å^2^)

**Table:**

	*x*	*y*	*z*	*U*_iso_*/*U*_eq_	
O1	0.34875 (17)	0.16856 (16)	0.5089 (3)	0.0305 (5)	
N1	0.62903 (19)	0.25690 (19)	0.5191 (3)	0.0229 (5)	
N2	0.6299 (2)	0.2508 (2)	0.3598 (3)	0.0291 (6)	
N3	0.4892 (2)	0.35743 (18)	0.5996 (3)	0.0255 (6)	
N4	0.5617 (2)	0.4320 (2)	0.6770 (4)	0.0378 (7)	
N5	0.5520 (2)	0.1924 (2)	0.7270 (3)	0.0236 (5)	
N6	0.5898 (2)	0.0891 (2)	0.7266 (3)	0.0296 (6)	
C1	0.5280 (2)	0.2497 (2)	0.5740 (3)	0.0212 (6)	
C2	0.7343 (3)	0.2658 (3)	0.3557 (4)	0.0336 (7)	
H2A	0.7610	0.2658	0.2591	0.040*	
C3	0.8001 (2)	0.2816 (2)	0.5100 (4)	0.0318 (7)	
H3A	0.8765	0.2933	0.5376	0.038*	
C4	0.7295 (3)	0.2762 (2)	0.6118 (4)	0.0303 (7)	
H4A	0.7475	0.2845	0.7259	0.036*	
C5	0.5010 (3)	0.5170 (3)	0.6921 (5)	0.0426 (9)	
H5A	0.5290	0.5831	0.7406	0.051*	
C6	0.3900 (3)	0.4977 (3)	0.6277 (5)	0.0394 (8)	
H6A	0.3311	0.5461	0.6252	0.047*	
C7	0.3848 (3)	0.3954 (3)	0.5699 (4)	0.0307 (7)	
H7A	0.3210	0.3574	0.5187	0.037*	
C8	0.6077 (3)	0.0592 (3)	0.8804 (4)	0.0330 (7)	
H8A	0.6344	−0.0098	0.9187	0.040*	
C9	0.5825 (3)	0.1410 (3)	0.9791 (4)	0.0379 (8)	
H9A	0.5884	0.1385	1.0927	0.045*	
C10	0.5474 (2)	0.2258 (3)	0.8775 (4)	0.0324 (7)	
H10A	0.5244	0.2946	0.9067	0.039*	
C11	0.4437 (2)	0.1869 (2)	0.4498 (4)	0.0252 (6)	
H11A	0.4249	0.2284	0.3478	0.030*	
H11B	0.4751	0.1169	0.4266	0.030*	
C12	0.3184 (3)	0.0580 (3)	0.5101 (5)	0.0384 (8)	
H12A	0.2517	0.0515	0.5511	0.058*	
H12B	0.3770	0.0171	0.5800	0.058*	
H12C	0.3057	0.0291	0.4000	0.058*	

### Atomic displacement parameters (Å^2^)

**Table:**

	*U*^11^	*U*^22^	*U*^33^	*U*^12^	*U*^13^	*U*^23^
O1	0.0238 (11)	0.0263 (11)	0.0426 (13)	−0.0028 (9)	0.0099 (9)	−0.0036 (9)
N1	0.0226 (13)	0.0254 (12)	0.0214 (13)	0.0009 (10)	0.0062 (10)	0.0004 (10)
N2	0.0335 (15)	0.0325 (14)	0.0239 (13)	0.0045 (11)	0.0115 (11)	0.0029 (11)
N3	0.0233 (12)	0.0213 (12)	0.0324 (13)	−0.0003 (10)	0.0072 (10)	−0.0024 (11)
N4	0.0282 (14)	0.0256 (14)	0.0600 (19)	−0.0051 (12)	0.0103 (13)	−0.0140 (13)
N5	0.0247 (12)	0.0237 (12)	0.0227 (12)	−0.0018 (10)	0.0058 (10)	−0.0001 (10)
N6	0.0359 (15)	0.0213 (13)	0.0294 (14)	0.0003 (10)	0.0025 (11)	0.0033 (10)
C1	0.0197 (14)	0.0219 (14)	0.0233 (15)	0.0004 (10)	0.0074 (12)	−0.0010 (11)
C2	0.0357 (17)	0.0306 (17)	0.0400 (19)	0.0029 (14)	0.0203 (15)	0.0051 (14)
C3	0.0222 (15)	0.0285 (15)	0.048 (2)	0.0000 (12)	0.0143 (15)	0.0000 (14)
C4	0.0257 (16)	0.0316 (17)	0.0334 (17)	0.0020 (13)	0.0057 (13)	−0.0024 (13)
C5	0.0370 (18)	0.0272 (18)	0.064 (2)	−0.0032 (14)	0.0130 (17)	−0.0149 (16)
C6	0.0308 (17)	0.0274 (17)	0.061 (2)	0.0062 (14)	0.0116 (16)	−0.0077 (15)
C7	0.0249 (15)	0.0287 (16)	0.0374 (17)	0.0024 (12)	0.0042 (13)	−0.0008 (13)
C8	0.0248 (16)	0.0364 (18)	0.0353 (18)	−0.0083 (14)	0.0009 (13)	0.0113 (15)
C9	0.0300 (17)	0.060 (2)	0.0246 (16)	−0.0035 (16)	0.0075 (13)	0.0056 (15)
C10	0.0260 (16)	0.0457 (19)	0.0282 (16)	−0.0028 (14)	0.0122 (13)	−0.0058 (14)
C11	0.0214 (15)	0.0256 (14)	0.0280 (15)	0.0008 (11)	0.0041 (12)	−0.0027 (12)
C12	0.038 (2)	0.0282 (17)	0.051 (2)	−0.0059 (14)	0.0126 (17)	0.0031 (16)

### Geometric parameters (Å, º)

**Table:**

O1—C11	1.411 (3)	C3—H3A	0.9500
O1—C12	1.423 (4)	C4—H4A	0.9500
N1—N2	1.356 (3)	C5—C6	1.405 (5)
N1—C4	1.357 (4)	C5—H5A	0.9500
N1—C1	1.448 (3)	C6—C7	1.355 (5)
N2—C2	1.335 (4)	C6—H6A	0.9500
N3—N4	1.363 (4)	C7—H7A	0.9500
N3—C7	1.365 (4)	C8—C9	1.394 (5)
N3—C1	1.454 (3)	C8—H8A	0.9500
N4—C5	1.323 (4)	C9—C10	1.369 (5)
N5—C10	1.354 (4)	C9—H9A	0.9500
N5—N6	1.364 (3)	C10—H10A	0.9500
N5—C1	1.452 (4)	C11—H11A	0.9900
N6—C8	1.327 (4)	C11—H11B	0.9900
C1—C11	1.534 (4)	C12—H12A	0.9800
C2—C3	1.402 (5)	C12—H12B	0.9800
C2—H2A	0.9500	C12—H12C	0.9800
C3—C4	1.369 (4)		
			
C11—O1—C12	114.0 (2)	N4—C5—H5A	124.0
N2—N1—C4	112.3 (2)	C6—C5—H5A	124.0
N2—N1—C1	121.0 (2)	C7—C6—C5	105.3 (3)
C4—N1—C1	126.7 (2)	C7—C6—H6A	127.3
C2—N2—N1	103.8 (3)	C5—C6—H6A	127.3
N4—N3—C7	111.9 (2)	C6—C7—N3	106.7 (3)
N4—N3—C1	118.8 (2)	C6—C7—H7A	126.7
C7—N3—C1	129.0 (2)	N3—C7—H7A	126.7
C5—N4—N3	104.2 (3)	N6—C8—C9	112.0 (3)
C10—N5—N6	112.0 (3)	N6—C8—H8A	124.0
C10—N5—C1	130.5 (3)	C9—C8—H8A	124.0
N6—N5—C1	117.4 (2)	C10—C9—C8	105.3 (3)
C8—N6—N5	104.1 (3)	C10—C9—H9A	127.4
N1—C1—N5	106.9 (2)	C8—C9—H9A	127.4
N1—C1—N3	109.8 (2)	N5—C10—C9	106.6 (3)
N5—C1—N3	109.0 (2)	N5—C10—H10A	126.7
N1—C1—C11	109.6 (2)	C9—C10—H10A	126.7
N5—C1—C11	110.1 (2)	O1—C11—C1	110.5 (2)
N3—C1—C11	111.3 (2)	O1—C11—H11A	109.5
N2—C2—C3	112.3 (3)	C1—C11—H11A	109.5
N2—C2—H2A	123.8	O1—C11—H11B	109.5
C3—C2—H2A	123.8	C1—C11—H11B	109.5
C4—C3—C2	104.4 (3)	H11A—C11—H11B	108.1
C4—C3—H3A	127.8	O1—C12—H12A	109.5
C2—C3—H3A	127.8	O1—C12—H12B	109.5
N1—C4—C3	107.1 (3)	H12A—C12—H12B	109.5
N1—C4—H4A	126.4	O1—C12—H12C	109.5
C3—C4—H4A	126.4	H12A—C12—H12C	109.5
N4—C5—C6	112.0 (3)	H12B—C12—H12C	109.5
			
C4—N1—N2—C2	0.6 (3)	N4—N3—C1—C11	−165.4 (3)
C1—N1—N2—C2	177.0 (3)	C7—N3—C1—C11	21.3 (4)
C7—N3—N4—C5	−1.0 (4)	N1—N2—C2—C3	−0.1 (3)
C1—N3—N4—C5	−175.4 (3)	N2—C2—C3—C4	−0.4 (4)
C10—N5—N6—C8	0.5 (3)	N2—N1—C4—C3	−0.9 (3)
C1—N5—N6—C8	178.0 (2)	C1—N1—C4—C3	−177.1 (3)
N2—N1—C1—N5	144.6 (2)	C2—C3—C4—N1	0.8 (3)
C4—N1—C1—N5	−39.6 (4)	N3—N4—C5—C6	0.9 (4)
N2—N1—C1—N3	−97.3 (3)	N4—C5—C6—C7	−0.5 (5)
C4—N1—C1—N3	78.6 (3)	C5—C6—C7—N3	−0.2 (4)
N2—N1—C1—C11	25.2 (3)	N4—N3—C7—C6	0.8 (4)
C4—N1—C1—C11	−158.9 (3)	C1—N3—C7—C6	174.4 (3)
C10—N5—C1—N1	115.6 (3)	N5—N6—C8—C9	−0.2 (3)
N6—N5—C1—N1	−61.3 (3)	N6—C8—C9—C10	−0.2 (4)
C10—N5—C1—N3	−3.0 (4)	N6—N5—C10—C9	−0.6 (3)
N6—N5—C1—N3	−179.9 (2)	C1—N5—C10—C9	−177.7 (3)
C10—N5—C1—C11	−125.4 (3)	C8—C9—C10—N5	0.4 (3)
N6—N5—C1—C11	57.7 (3)	C12—O1—C11—C1	−124.7 (3)
N4—N3—C1—N1	−43.9 (3)	N1—C1—C11—O1	173.5 (2)
C7—N3—C1—N1	142.8 (3)	N5—C1—C11—O1	56.2 (3)
N4—N3—C1—N5	72.9 (3)	N3—C1—C11—O1	−64.8 (3)
C7—N3—C1—N5	−100.4 (3)		

### Hydrogen-bond geometry (Å, º)

**Table:**

*D*—H···*A*	*D*—H	H···*A*	*D*···*A*	*D*—H···*A*
C5—H5*A*···N2^i^	0.95	2.51	3.453 (4)	171
C9—H9*A*···N2^ii^	0.95	2.61	3.433 (4)	145
C4—H4*A*···O1^iii^	0.95	2.53	3.444 (4)	162

Symmetry codes: (i) *x*, −*y*+1, *z*+1/2; (ii) *x*, *y*, *z*+1; (iii) *x*+1/2, −*y*+1/2, *z*+1/2.
