# Supplementary figures and images for: Crystal structure of 1-meth­oxy-2,2,2-tris­(pyrazol-1-yl)ethane
Source: Acta Crystallogr Sect E Struct Rep Online. 2014 Aug 23;70(Pt 9):o1047–8. doi: 10.1107/S1600536814018789 (PMC4186097; doi:10.1107/S1600536814018789)

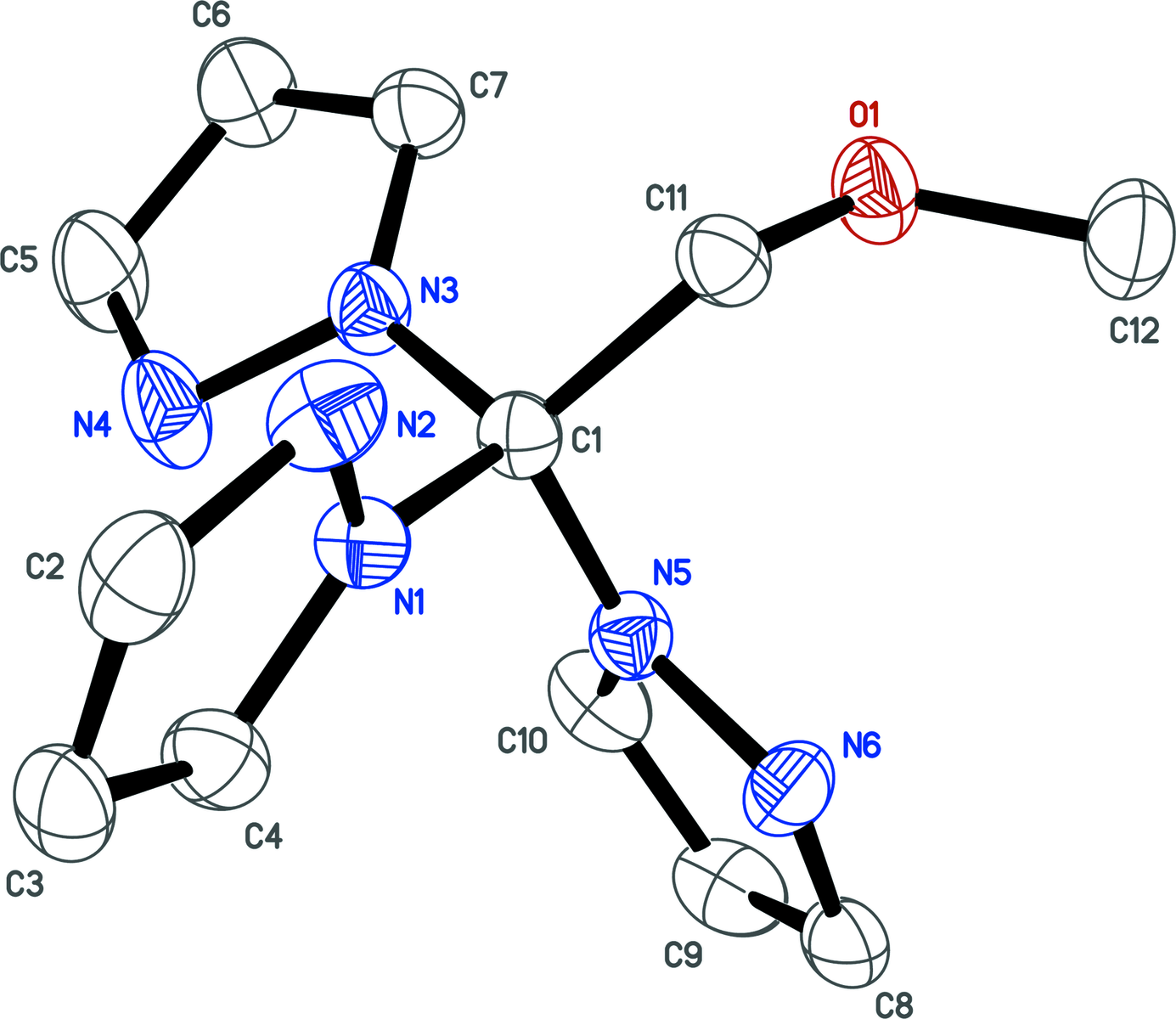

Supplement: Supplementary file 4 [file e-70-o1047-fig1.tif]
